# A meta-analytic review of prevalence for Brugada ECG patterns and the risk for death

**DOI:** 10.1097/MD.0000000000005643

**Published:** 2016-12-16

**Authors:** Xiao-Qing Quan, Song Li, Rui Liu, Kai Zheng, Xiao-Fen Wu, Qiang Tang

**Affiliations:** a Department of Geriatrics; b Second Clinical School, Tongji Hospital; c Department of Pharmacology, School of Basic Medicine, Tongji Medical College, Huazhong University of Science and Technology, Wuhan, Hubei, China.

**Keywords:** Brugada ECG pattern, Brugada syndrome, risk ratio, sudden cardiac death, ventricular fibrillation

## Abstract

**Background:**

The prevalence of Brugada ECG pattern (BrEP) is different in different regions, and its mean prevalence over the world is unknown. The risk of people with BrEP for death remains unknown. We performed a meta-analysis to determine the prevalence of BrEP and risk ratio (RR) for death.

**Methods:**

Relevant studies published between July 1, 2000 and August 20, 2016, which contain prevalence and RR for all-cause death and cardiac death, were included. The prevalence and RR are analyzed using meta-analysis.

**Results:**

We finally retrieved 24 studies of the prevalence for BrEP and 5 studies of the RR for all-cause death and cardiac death. The worldwide mean prevalence of BrEP is 0.4%, with highest in Asia (0.9%) and lowest in North America (0.2%). Additionally, the mean prevalence in male is 0.9%, whereas it is 0.1% in female. The RR of BrEP for all-cause death is 0.78 (95% confidence interval 0.45–1.37), and for cardiac death it is 0.92 (95% confidence interval 0.23–3.66).

**Conclusion:**

The prevalence of BrEP is about 0.4% around the world with different prevalence in region and sex. Our study shows that BrEP may not be taken as a predictor of all-cause death and cardiac death.

## 1 Introduction

Since 1992, when Brugada syndrome (BrS) was first described,^[1]^ the BrS has been universally recognized as a cause of arrhythmia, syncope, ventricular fibrillation (VF), and sudden cardiac death (SCD) without structural heart disease.^[2]^ BrS is responsible for up to 20% of SCDs (worldwide) in patients with structurally normal hearts.^[3,4]^ The prevalence of Brugada ECG pattern (BrEP) is different in region and sex, and such differences range greatly with a greater prevalence in Asians and in men.^[5]^ However, its mean prevalence over the world is unknown.

The typical ECG of BrS is known as the BrEP, which is characterized by right bundle-branch block and ST-segment elevation in the right precordial leads (V1–V3).^[2]^ Based on the differences of the ECG patterns (eg, the extent of ST-segment elevation), BrEP can be divided into 3 types.^[6]^ BrS might show a variable ECG presentation that includes the 3 types at different times.^[2,5,6]^ According to the 2013 consensus report, the type I Brugada ECG pattern is the main criterion for the diagnosis of the BrS, whereas types II and III are at low risk.^[7]^ It is also known that some physiologic causes such as normal variant, incomplete right bundle-branch block, and pathologic causes such as pulmonary hypertension and hyperkalemia can result in BrEP.^[8,9]^ The diagnosis of BrS has been suggested to combine the ECG patterns and the clinical symptoms.^[5,6]^

The risk for people with BrEP remains controversial. The incidence of VF, SCD, or syncope differs greatly, especially between patients with and without a history of SCD or syncope, and may be relative high in Asian patients.^[5]^ This study aims to identify regional prevalence for BrEP and the risk of death in people with BrEP worldwide.

## 2 Methods

This systematic review and meta-analysis was launched based on the Preferred Reporting Items for Systematic Reviews and Meta-Analyses (PRISMA) statement. There are no ethical issues involved in our study as our data were based on published studies.

### 2.1 Definition of Brugada ECG

Brugada ECG has been divided into 3 types, defined as follows^[6]^:

Type I: Prominent coved ST-T segment with a J-wave amplitude, or ST-segment elevation ≥2 mm at the peak with a negative T-wave without isoelectric separation followed.
Type II: High take-off ST segment with a J-wave amplitude ≥2 mm followed by gradually decreasing ST-segment elevation (≥1 mm above the baseline), and a biphasic or positive T-wave that arises saddle-back configuration.
Type III: Right precordial ST-segment elevation <1 mm of a saddle-back type or/with coved type.

### 2.2 Study selection

We performed a comprehensive and systematic search of retrospective, prospective, randomized, or nationwide studies in PubMed, EMBASE, Medline, and The Cochrane Library using terms “Brugada ECG patterns,” “Brugada syndrome,” “prevalence of Brugada,” “death,” “prognosis,” “mortality,” and “meta-analysis.” Only the studies whose patients have BrEP will be included in our study. As the prevalence of BrEP has been estimated about 0.5%,^[10]^ we only choose the studies whose total number of the patients is more than 200. And, as we hope to find out the regional prevalence of BrEP, the studies are also limited to regional investigations which were made in a random group of people.

**Figure 1:**
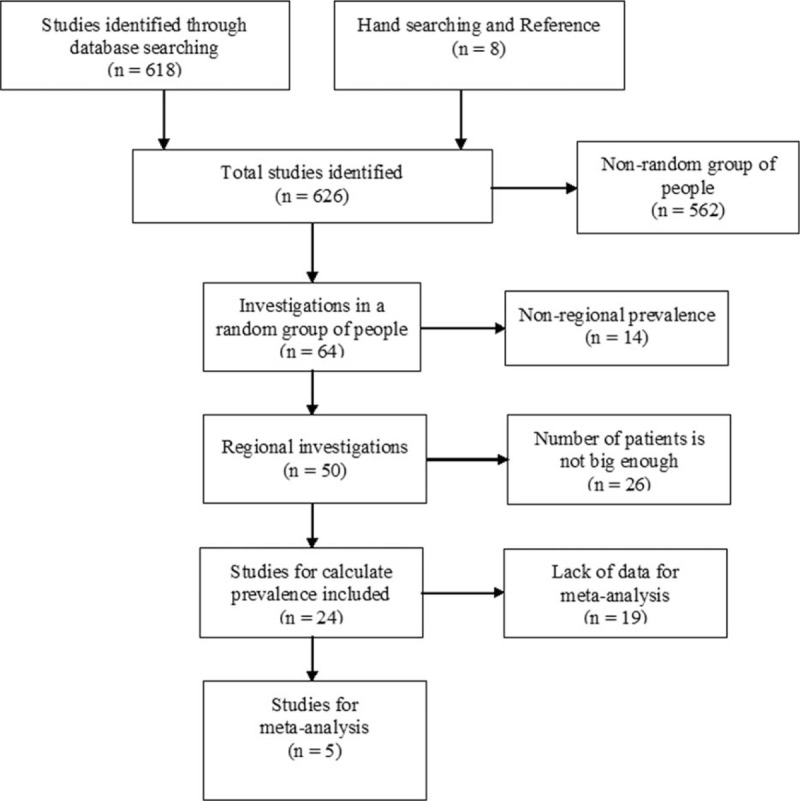
Flow diagram of literature search and study selection.

The detailed selection process is listed in Fig. 1. We searched 626 studies totally, but only 24 studies can be included for the calculation of prevalence. Only 5 of the 24 studies can be used for meta-analysis of risk ratio (RR). According to the endpoint, we performed meta-analysis of the risk of BrEP for all-cause death and cardiac death.

### 2.3 Data extraction

From each retrieved study, the following data were extracted: name of the lead investigator and year of the paper, the region or country where the study was performed, sample size, proportions of men and women, the mean age, follow-up time, total prevalence, male prevalence and female prevalence, types of BrEP, the primary and secondary endpoints, RR, or hazard ratio (HR) with 95% confidence interval (CI).

The following criteria are used for the assessment of the risk of bias and methodological quality: definition of BrEP, sample size, study design, inclusion criteria, sex ratio, and the duration of the follow-up.

### 2.4 Data synthesis

With the basis of random-effects model developed by DerSimonian and Laird, the Begg test, which has been demonstrated with Cochran *Q* test and *I*^2^ statistic of the homogeneity of the results, was used for the evaluation of publication bias. And finally, we omitted each study 1 at a time to perform sensitivity analysis to examine the influence of each study on the pooled estimation. RR and 95% CI were calculated or recalculated for each study.^[11]^

Chi-square-based *Q* test was performed for the analysis of the heterogeneity of reported prevalence with 95% CI.^[12,13]^ After the heterogeneity test, we found important variations between studies. So, with the purpose of getting better results, we used the random-effects model^[14,15]^ for the estimation of the prevalence of BrEP.

The results are shown in forest plots (the point estimations and their 95% CI). Statistical significance was set at a *P* value <0.05, and all tests performed were 2-sided.^[16]^ Meta-analysis was performed using Stata version 12 (StataCorp LP, College Station, TX).

## 3 Results

After search and selection, we finally found 24 articles to be included in our study (Table 1).^[17–40]^ Only 2 studies^[30,32]^ have included merely type I BrEP, the rest studies have included all the 3 types of ECG patterns. Eleven of the 24 studies^[21–23,25,26,28–30,34,38,40]^ have also studied the sex differences for the prevalence of BrEP. Five studies^[21,27,33,38,40]^ have studied the RR of all-cause death and cardiac death for the patients with BrEP. All the 5 studies^[21,27,33,38,40]^ we used for meta-analysis of RR included all the 3 types ECG patterns.

**Table 1:**
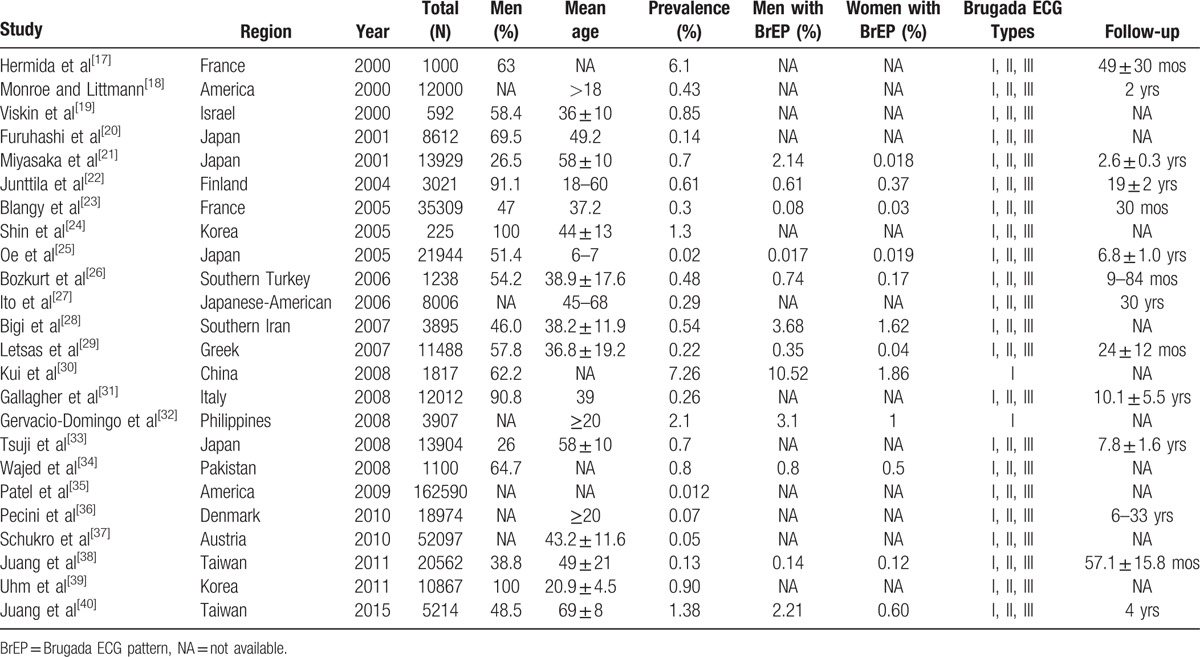
Summary characteristics of prevalence of BrEP in each study.

**Figure 2:**
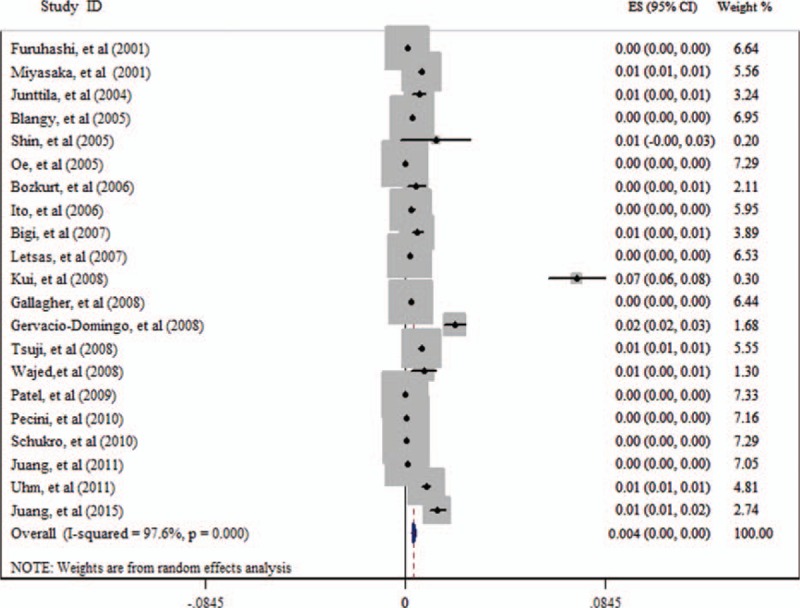
Result of the mean global prevalence of Brugada ECG pattern (BrEP).

All these studies cover an extensive area all over the world, including Asia, Europe, and North America. According to these studies, the mean prevalence of BrEP across the world is 0.4% (Fig. 2, Table 2). The results of regional prevalence of BrEP are presented in Table 2. The prevalence of BrEP is 0.9%, 0.3%, and 0.2% in Asia,^[19–21,24,25,28,30,32–34,38–40]^ Europe,^[17,22,23,26,29,31,36,37]^ and North America,^[18,27,35]^ respectively (Table 2).

**Table 2:**
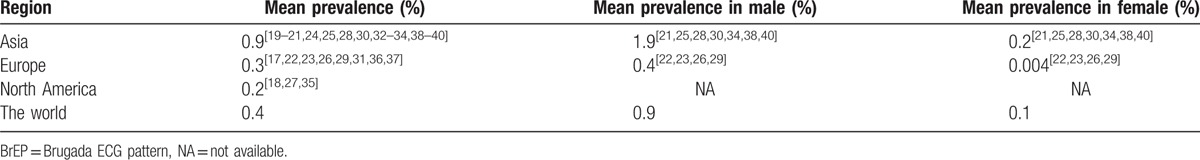
Mean prevalence and mean prevalence of different sex of BrEP in each region.

Eleven articles^[21–23,25,26,28–30,34,38,40]^ have studied the sex differences of the prevalence of BrEP (Table 2). The mean prevalence of BrEP in male is 0.9%, whereas that in female is 0.1% in the world. Additionally, the prevalence in male is always higher than that in female in all regions. The prevalence of both male (1.9%) and female (0.2%) is the highest in Asia.

**Figure 3:**
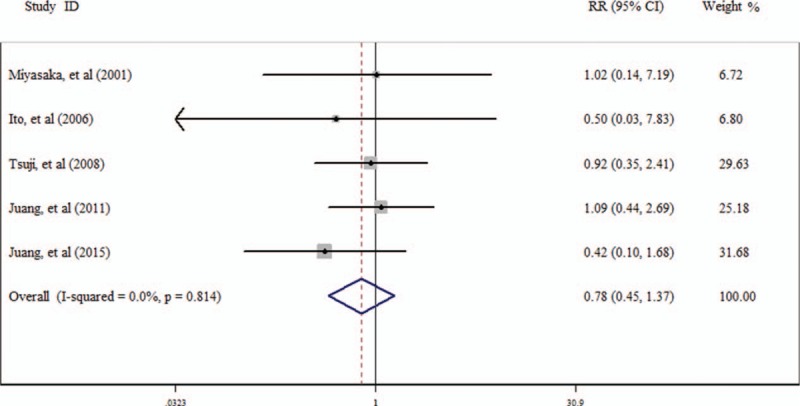
Result of the risk ratio (RR) of Brugada ECG pattern (BrEP) for all-cause death.

**Figure 4:**
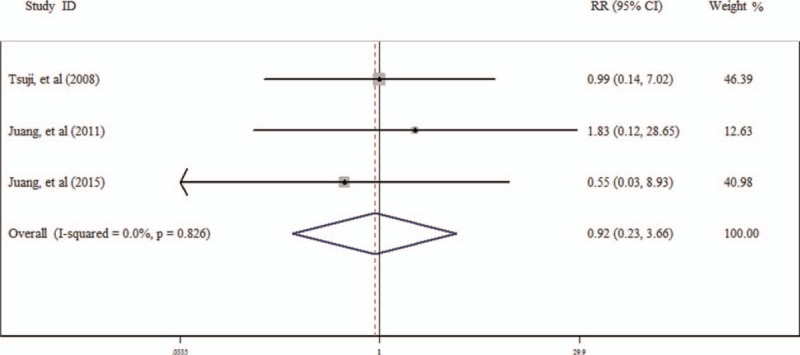
Result of the risk ratio (RR) of Brugada ECG pattern (BrEP) for cardiac death.

Five studies^[21,27,33,38,40]^ have studied the RR of all-cause death and cardiac death for the people with BrEP (Table 3). The RR of BrEP for all-cause death is 0.78 (95% CI 0.45–1.37; Fig. 3), and for cardiac death it is 0.92 (95% CI 0.23–3.66; Fig. 4).

**Table 3:**
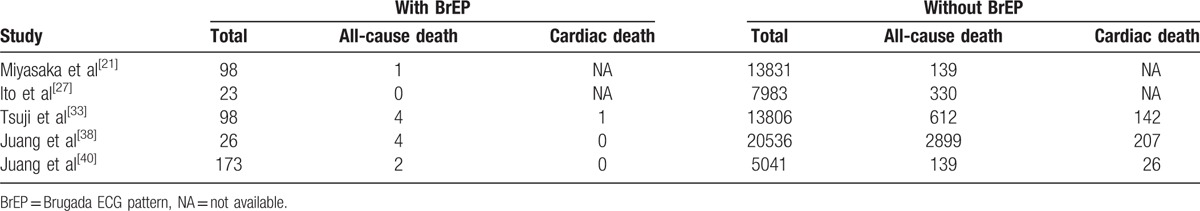
Summary characteristics of death ratio of people with BrEP and without BrEP.

## 4 Discussion

This study yielded the following novel findings: the prevalence of BrEP is about 0.4% around the world with different prevalence in region and sex; BrEP may not be taken as a predictor of all-cause death and cardiac death.

The right bundle-branch block and ST-segment elevation in the right precordial leads (V1–V3) are the main characteristics of BrEP.^[2]^ BrS has been considered as a cause of arrhythmia, syncope, VF, and SCD without structural heart disease.^[2]^ According to the 2013 consensus report, the finding of type I Brugada ECG pattern is the basis of the diagnosis of BrS.^[7]^ However, some experts have suggested that either symptoms such as agonal nocturnal respiration, cardiac arrest, unexplained syncope, and documented ventricular tachycardia/VF, or positive family history such as diagnosed BrS in a first-degree relative or unexplained SCD <45 years be included in the diagnostic criteria.^[5]^ If a type I Brugada ECG pattern is observed without any clinical criteria, this should be referred to as “idiopathic BrEP” and not as BrS.^[41,42]^

The present study shows that the prevalence of BrEP is significantly different between Asia, Europe, and North America (Table 2). It shows that the racial differences, geographical differences, and regional tradition may play roles in the prevalence of BrEP. The prevalence in Asia is much higher than that in the other places of the world, over 4 times as much as it is in North America. And the prevalence in Europe is close to the average level of the world, with a prevalence of 0.3%. Our study shows that men are over 4 times easier to get BrEP than women (Table 2). These results are consistent with previous studies,^[5,6,43]^ and we confirm the existence of the regional and sex difference in prevalence of BrEP.

Our study shows that the RR of BrEP for all-cause death and cardiac death are 0.78 and 0.92, respectively, for people with BrEP. It shows that BrEP may not be taken as a predictor of all-cause death and cardiac death. However, according to the previous observations, the people with BrEP are at high risk of sudden death.^[44–47]^ These studies are mainly hospital-based studies. The people included in these studies have symptoms like syncope and VF, or history of SCD. The studies we included are community-based studies^[21,40]^ and population-based studies,^[27,33,38]^ in which the participants may not have symptoms or history as mentioned above.

Another possible reason for the negative result of present study is that all the studies^[21,27,33,38,40]^ we used for meta-analysis of RR included all the 3 types of ECG patterns. Previous studies showed that type I BrEP is at high risk, whereas types II and III are at low risk.^[2,6,42]^ Further studies are needed to estimate type I BrEP for all-cause death and cardiac death.

### 4.1 Limitations

Our study has the following limitations:

1. The subjects in our study are mostly from Asia and their parameters could not represent the state of the whole population worldwide.
2. We included the people with BrEP into our study, and the symptoms such as cardiac arrest and unexplained syncope were not considered as criteria. Further studies based upon both BrEP and clinical symptoms, or history, are needed.
3. Most of the studies in our research included all the 3 ECG patterns. Further studies which focus on type I BrEP are needed.

## 5 Conclusions

The prevalence of BrEP in the general population is about 0.4%, whereas it differs extensively in terms of region and sex. BrEP may not be taken as a predictor of all-cause death and cardiac death.
